# A dual-antigen malaria vaccine targeting Pb22 and Pbg37 was able to induce robust transmission-blocking activity

**DOI:** 10.1186/s13071-023-06071-x

**Published:** 2023-12-14

**Authors:** Wenyan Gao, Yue Qiu, Liying Zhu, Xinxin Yu, Fan Yang, Muyan Chen, Gang He, Yinjie Liu, Liwang Cui, Fei Liu, Xiaotong Zhu, Yaming Cao

**Affiliations:** 1 Department of Immunology, College of Basic Medical Sciences, China Medical University, No.77 Puhe Road, Shenyang, 110122 Liaoning People’s Republic of China; 2 Department of Obstetrics, The First Affiliated Hospital of China Medical University, NO. 155, Nanjing Street, Shenyang, 110001 Liaoning People’s Republic of China; 3 Department of Cardiovascular Ultrasound, The First Hospital of China Medical University, Shenyang, 110001 Liaoning China; 4 Department of Internal Medicine, Morsani College of Medicine, University of South Florida, Tampa, FL 33612 USA

**Keywords:** Malaria, Transmission reducing activity, Dual antigen vaccine, Sexual stage development

## Abstract

**Background:**

Despite years of effort to develop an effective vaccine against malaria infection, a vaccine that provides individuals with sufficient protection against malaria illness and death in endemic areas is not yet available. The development of transmission-blocking vaccines (TBVs) is a promising strategy for malaria control. A dual-antigen malaria vaccine targeting both pre- and post-fertilization antigens could effectively improve the transmission-blocking activity of vaccines against the sexual stages of the parasite.

**Methods:**

A chimeric recombinant protein Pb22-Pbg37 (*Plasmodium berghei* 22-*P. berghei* G37) composed of 19–218 amino acids (aa) of Pb22 and the N-terminal 26–88 aa of Pbg37 was designed and expressed in the *Escherichia coli* expression system. The antibody titers of the fusion (Pb22-Pbg37) and mixed (Pb22+Pbg37) antigens, as well as those of Pb22 and Pbg37 single antigens were evaluated by enzyme-linked immunosorbent assay. Immunofluorescence and western blot assays were performed to test the reactivity of the antisera with the native proteins in the parasite. The induction of transmission-blocking activity (TBA) by Pb22-Pbg37 and Pb22+Pbg37 were evaluated by in vitro gametocyte activation, gamete and exflagellation center formation, ookinete conversion, and in the direct mosquito feeding assay.

**Results:**

The Pb22-Pbg37 fusion protein was successfully expressed in vitro. Co-administration of Pb22 and Pbg37 as a fusion or mixed protein elicited comparable antibody responses in mice and resulted in responses to both antigens. Most importantly, both the mixed and fusion antigens induced antibodies with significantly higher levels of TBA than did each of the individual antigens when administered alone. In addition, the efficacy of vaccination with the Pb22-Pbg37 fusion protein was equivalent to that of vaccination with the mixed single antigens.

**Conclusions:**

Dual-antigen vaccines, which expand/lengthen the period during which the transmission-blocking antibodies can act during sexual-stage development, can provide a promising higher transmission-reducing activity compared to single antigens.

**Graphical Abstract:**

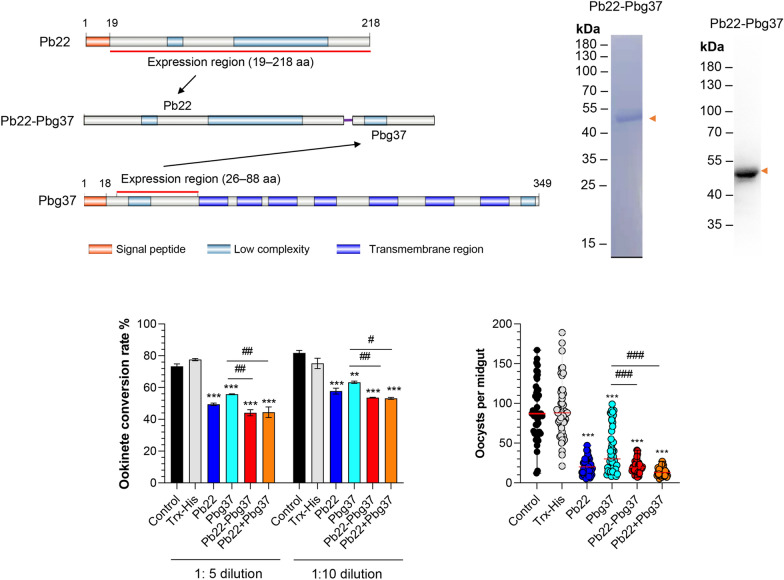

**Supplementary Information:**

The online version contains supplementary material available at 10.1186/s13071-023-06071-x.

## Background

Malaria is the leading cause of morbidity and mortality in tropical and subtropical countries, with approximately 619,000 deaths reported by WHO in 2021 [1]. The emergence and spread of resistance to the first-line drug artemisinin and other insecticides have increased malaria incidence in the last few years, making the need for an effective vaccination more urgent than ever [2]. To date, one of the most efficacious vaccines against malaria is the RTS,S/AS01 vaccine that targets the pre-erythrocyte stage [3]. However, this vaccine only gives partial protection against *Plasmodium falciparum*, and its efficacy wanes rapidly in the first 6 months [4]. The developmental switch into the sexual stage is responsible for malaria transmission in endemic areas. Indeed, the successful elimination of malaria can only be achieved by interrupting or reducing transmission in endemic areas until no parasites remain [5, 6]. Therefore, the development of a vaccine that interrupts/stops the development of the sexual stage of the malaria parasite may be highly beneficial in malaria control.

Vaccines targeting the sexual stage (transmission-blocking vaccine [TBV]) are thought to be capable of breaking the cycle of *Plasmodium*. The principle of malaria TBVs is that antibodies raised against the sexual-stage surface antigen of the malaria parasite could arrest the development of the sexual stage of the parasite in the mosquito’s midgut [7, 8]. Due to a lower immune pressure during evolution, TBV candidates tend to be less polymorphic than antigens expressed in the pre-erythrocyte and asexual erythrocyte stages. TBVs are also thought to help prevent the spread of emerging drug-resistant parasites [9, 10]. Several promising candidates have been extensively investigated for TBV development, including the pre-fertilization antigens P230, P48/45, HAP2 and P230p, and the post-fertilization antigens P25 and P28 [11–16]. However, success in clinical trials has been limited, and none of these TBV candidates were able to induce complete blocking of malaria transmission [10]. It is possible that antigens other than these candidates contribute to protective immunity, which prompted us to identify additional TBV candidates and develop novel immunization methods, with the aim to provide a powerful tool to prevent malaria transmission.

Encouragingly, with science progressing into the -omics era, many more potential TBV antigens have been identified. Previous work from our group using murine *Plasmodium berghei* models have evaluated the transmission-blocking activity (TBA) of several antigens, including *Plasmodium berghei* 22 (Pb22), *P. berghei* G37 (Pbg37), *P. berghei* pleckstrin homology (PbPH), putative secreted ookinete protein 26 (PSOP26), *P. berghei* G-Protein-Coupled Receptor (PbGPR180), *P. berghei* 51 (Pb51) and quiescin sulfhydryl oxidase (QSOX) [17–23]. Among these antigens, Pbg37 and Pb22 are expressed at both the pre- and post-fertilization phases. They are also surface antigens of gametes and developing ookinetes. The results of one study showed that Pbg37 is dominantly expressed in the gametocyte stage and known to be highly conserved between *Plasmodium* spp. and essential for male-specific gametogenesis [18]. In the same study, the vaccine, which targeted the N-terminal of Pbg37, significantly decreased male gametogenesis [18]. In comparison, the major function of Pb22 is to regulate ookinete formation and ookinete-oocyst transition [17]. The antisera against Pb22 and *Plasmodium vivax* 22 (Pv22) were observed to induce significant functional activity toward reducing oocyst intensity and mosquito infectivity [17, 24]. Therefore, it would appear that both Pbg37 and Pb22 are promising candidate TBV antigens and should be investigated further.

*Plasmodium* dual-antigen vaccines that target both asexual and sexual stage antigens of the malaria parasites have been shown to be well tolerated, safe and immunogenic in clinical trials performed in malaria endemic areas; those tested to date include the GMZ2.6c malaria vaccine, which contains merozoite surface protein 3 (MSP3), glutamate-rich protein (GLURP) and Pfs48/45 [25, 26]. A study on the development of a pre-erythrocyte-stage vaccine showed that combining TRAP and RTS,S-like vaccine R21 in a single formulation significantly enhanced protective efficacy compared to single-component vaccines in a murine model [27]. Two studies additionally showed that the protective immunity induced by dual-antigen malaria vaccines PfCSP-Pfs25 or Pvs25-PvCSP was able to provide a promising high level of transmission-reducing activity (TRA; > 99% for PfCSP-Pfs25; 82% for Pvs25-PvCSP) [28, 29]. Comparable to the results of these reports, in our recent work, we showed that fusing or mixing Pbg37 with ookinete surface antigen PSOP25 for immunization was able to enhance the TRA efficiency afforded by Pbg37 alone [30]. These findings provide support that dual-antigen malaria vaccines, which provide greater overall protection, cost-effectiveness and durability than a single-stage vaccine, will be a powerful tool for malaria control. We therefore set out to test the combination of Pb22 and Pbg37 antigens on the TRA of malaria parasites.

In the present study, two sexual-stage antigens, Pb22 and Pbg37, were evaluated for applicability as a bivalent TBV using the *P. berghei* rodent malaria model. Under the immunization conditions used, there was no obvious immune interference between the two selected antigens. Moreover, the Pb22 and Pbg37 antigens, used either as a mixed (Pb22+Pbg37) or as a fusion (Pb22-Pbg37) antigen for immunization, could elicit a significantly higher TRA than the same two antigens immunized separately.

## Methods

### Mice, parasites, and mosquitoes

Six-week-old female BALB/c mice were purchased from Beijing Animal Institute (Beijing, China) and used for propagating, transfecting and cloning parasites and for mosquito blood-feeding. The  *P. berghei* ANKA 2.34 line was used in this study. Female *Anopheles stephensi* mosquitoes (Hor strain) were fed on a 10% (w/v) glucose solution and maintained at 25 °C (pre-infection) or 19 °C (post-infection) and 50–80% humidity, under a 12/12-h light/dark cycle. All animal experiments were performed in accordance with the welfare and ethical review standards of China Medical University.

### Recombinant protein expression, purification and mice immunizations

To generate a Pb22 and Pbg37 fusion protein (rPb22-Pbg37), we fused a gene fragment of Pb22 corresponding to amino acids (aa) 19–218 and a gene fragment of Pbg37 corresponding to aa 26–88, with a flexible (GGGGS)_3_ spacer in between, by overlapping PCR (primers are listed in Additional file 1: Table S1). The purified PCR product was subcloned into a pET-32a(+) vector (Novagen, Darmstadt, Germany) with the ClonExpress® II One Step Cloning Kit (Vazyme, Nanjing, China) to generate the pET-32a-Pb22-Pbg37 plasmid. The pET-32a-Pb22-Pbg37 plasmid was transformed into *Escherichia coli* Rosetta-gami 2 (DE3), and protein expression was induced with 1 mM IPTG. The pET-32a(+) plasmid used for recombinant Pb22 (rPb22; aa 19–218 of Pb22 protein) and recombinant Pbg37 (rPbg37; 19–218 aa of Pbg37 protein) expression was construct by annealed PCR using specific primers, and the recombinant proteins were produced as described above (Additional file 1: Table S1). The expressed polyhistidine-tagged (His-tagged) recombinant proteins were affinity purified using Pierce™ Ni–NTA (Thermo Fisher Scientific, Waltham, MA, USA), followed by dialysis in 0.1 M phosphate-buffered saline (PBS) at 4 °C overnight.

The mouse anti-Pb22-Pbg37 and anti-Pb22+Pbg37 sera were raised as previously described [20]. Briefly, groups of 10 female BALB/c mice, 6–8-weeks-old, were immunized subcutaneously with the emulsified product of recombinant protein (50 μg per mouse for rPb22 and rPbg37, and rPb22-Pbg37 groups; 50 µg rPb22 + 50 µg rPbg37 per mouse for the rPb22+rPbg37 group) and complete Freund’s adjuvant (Sigma-Aldrich, St. Louis, MO, USA). Negative control mice were immunized with either PBS or thioredoxin (Trx)-His recombinant protein emulsified in the same adjuvant. Immunizations were enhanced (boosted) twice with recombinant proteins emulsified in incomplete Freund’s adjuvant (Sigma-Aldrich) at 2-week intervals. To obtain the antisera, blood was collected and allowed to clot at room temperature 10 days after the final immunization and stored at − 80 °C for the subsequent trials.

### Enzyme-linked immunosorbent assay

The specific antibody titers of Pb22-Pbg37 and Pb22+Pbg37, respectively, were quantified by enzyme-linked immunosorbent assay (ELISA) as described previously [31]. The rPb22 or rPbg37 antigen was produced using an *Escherichia coli* expression system. For the ELISA, 96-well microplates precoated with 10 μg/ml of rPb22 or rPbg37 protein were blocked with 1% bovine serum albumin (BSA) in PBS and then incubated with 100 μl serial dilutions of antisera (dilutions: 1:1000 to 1:128,000), as well as with negative control (anti-Trx-His sera). After three washes with 200 μl PBS containing 0.02% Tween-20 (PBS-T), horseradish peroxidase (HRP)-conjugated goat anti-mouse immunoglobulin G (IgG) antibodies (dilution: 1:5000) were added to the plates and the plates incubated for 1 h at 37 °C. The plates were then washed 6 times, and 100 μl of tetramethylbenzidine (TMB; Sigma-Aldrich) was added and the plates incubated in the dark for 5 min. Finally, the reaction was terminated with 50 μl of 2N H_2_SO_4_, and each plate was read at 450 nm using a Bio-Rad ELISA microplate reader (Bio-Rad Laboratories, Hercules, CA, USA).

### Western blot analysis

To detect the expression of Pb22-Pbg37, 5-μg aliquots of the purified recombinant proteins were boiled and separated by electrophoresis in 10% sodium dodecyl sulfate–polyacrylamide gels (SDS-PAGE) under reducing conditions, and the protein products transferred to polyvinylidene fluoride (PVDF) membranes (Bio-Rad Laboratories). The membranes were blocked with 5% skim milk at room temperature for 2 h, and then probed with anti-His monoclonal antibody (mAb; dilution: 1:5000; Thermo Fisher Scientific) for 2 h. To determine the specificity of the anti-sera, we purified the gametocytes and ookinetes as described previously [21]. Briefly, for gametocyte purification, the parasite-infected mice were treated with 20 mg/l sulfadiazine (Sigma-Aldrich) for 48 h to eliminate asexual-stage parasites, followed by separation on a 48% Nycodenz/RPMI 1640 culture medium gradient. For ookinetes, infected blood was harvested, cultured in an ookinete culture medium (RPMI 1640 supplemented with 50 mg/l penicillin, 50 mg/l streptomycin, 20% [vol/vol] FCS, 6 U/ml heparin; pH 8.0) at 19 °C for 24 h, and separated on a 62% Nycodenz density gradient medium [21]. A total of 50 μg of lysates from either the purified gametocytes or ookinetes was then electrophoresed on 10% SDS-PAGE gels and transferred to a PVDF membrane. After three washings, the membranes were probed with antisera against Pb22-Pbg37 or against Pb22+Pbg37, for 2 h at room temperature. The membranes were washed 3 times and incubated with HRP-conjugated goat anti-mouse IgG antibodies (Thermo Fisher Scientific) for 2 h at room temperature. The blots were then visualized with a Pierce™ ECL Western Blotting Substrate Kit (Thermo Fisher Scientific). Protein loading was estimated using the anti-rHsp70 sera (Hsp70; PBANKA_0711900) produced in the laboratory.

### Immunofluorescence assay

The specificity of antisera against the Pb22-Pbg37 and Pb22+Pbg37 recombinant proteins was analyzed by immunofluorescence assay (IFA) as described previously [32]. Briefly, the *P. berghei* parasites were fixed with 4% paraformaldehyde and 0.0075% glutaraldehyde (Sigma-Aldrich) in PBS for 30 min at room temperature (RT). The cells were then permeabilized with 0.1% (*v*/*v*) Triton X-100, followed by neutralization with 0.1 mg/ml of sodium borohydride. After blocking with 5% skim milk, the parasites were incubated with sera from immunized mice (dilution: 1:500) for 1 h, then co-labeled with rabbit antisera against P47 (dilution: 1:500), α-tubulin II (dilution: 1:500) or Pbs25 (dilution: 1:500) as stage-specific markers for female gametocytes/gametes, male gametocytes/gametes and zygotes/ookinetes, respectively, followed by three washing steps in PBS. The polyclonal antibodies against P47, α-tubulin II and Pbs25 were made in our laboratory [30]. Alexa Fluor 488-conjugated goat anti-mouse IgG antibodies (dilution: 1:500; Invitrogen, Thermo Fisher Scientific) and Alexa Fluor 555-conjugated goat anti-rabbit IgG antibodies (dilution: 1:500; Invitrogen, Thermo Fisher Scientific) were used as secondary antibodies. Negative controls were ookinetes incubated with the secondary antibodies alone, or ookinetes incubated with the anti-Trx-His serum. The samples were mounted with ProLong Diamond Antifade Mountant with DAPI (Thermo Fisher Scientific) and a coverslip, then sealed with nail polish before visualization. Fluorescence images of *P. berghei* parasites were observed and captured using a Leica STELLARIS 5 fluorescence confocal laser scanning microscope (Leica Microsystems, Wetzlar, Germany) and processed by Image J software.

### Transmission-blocking analysis

Anti-sera generated against the recombinant proteins (Pb22-Pbg37 and Pb22+Pbg37) were utilized in Transmission-blocking assays. For the in vitro assay, mice pre-treated with phenylhydrazine (PHZ) were infected intravenously (iv) with 1 × 10^7^ *P. berghei*-infected red blood cells (iRBC). At 3 days post-infection (dpi), parasitemias were determined, following which 10 μl of blood was collected from each of the appropriate hosts and added to 40 μl ookinete culture medium containing antisera against Pb22, Pbg37, Pb22-Pbg37, and Pb22+Pbg37 or Trx-His (negative control) at final dilutions of 1:5, respectively. The percentage of gametocyte activation, gametocyte-forming gametes and exflagellation centers were assessed as described previously [33]. Briefly, 10 μl of infected blood containing equal numbers of mature gametocytes was added to 40 μl ookinete medium at 25 °C for 30 min, and the male and female gametocytes/gametes were stained with anti-tubulin II/fluorescein isothiocyanate (FITC)-conjugated Ter-119 or anti-Pbg377/FITC-conjugated Ter-119 antibodies, respectively. Male and female gametes were specified by positive anti-tubulin II or Pbg377 fluorescence signals and a negative Ter-119 fluorescence signal. The male gametocytes were considered not activated if the nucleus signals had not divided at 25 °C for 8 min. The number of exflagellation centers formed was quantified 3 days after the iv infection by adding 10 μl of gametocyte-infected blood (the total number of gametocytes in 10 μl of infected tail blood was adjusted to equal numbers between each group) to 40 μl of ookinete medium and counting under a phase-contrast microscope at ×40 after a 15-min incubation at 25 °C.

Ookinete conversion rates were monitored as previously described [34]. Briefly, in vitro cultivated ookinetes were incubated with a mouse anti-Pbs21 mAb, and the conversion rate was calculated as the percentage of Pbs21-positive ookinetes to Pbs21-positive macrogametes and ookinetes. For the direct mosquito feeding assays (DFA), 1 × 10^7^ *P. berghei* iRBC were injected into mice pretreated with PHZ as described in section Transmission-blocking analysis. At 3 dpi, the mice with similar parasitemia (parasites per 2000 RBCs) and gametocytemia (mature gametocytes per 10,000 RBCs) were selected for DFA analysis. Briefly, the mice were injected iv. with 500 μl of antisera generated against rPb22, rPbg37, rPb22-Pbg37 and rPb22+Pbg37, respectively, anesthetized and were exposed for 1 h to *An. stephensi* mosquitoes (*n* = 80 per mice) pre-starved for 12 h. The unfed mosquitoes were removed. Mosquito transmission was assessed by dissection of ~ 50 mosquitoes in each group on day 10 post-feeding, which were analyzed for the oocyst infection prevalence and oocyst infection intensity by 0.5% Mercurochrome staining.

### Statistical analysis

All statistical analyses were performed using GraphPad Prism 9.0 software (GraphPad Software Inc., San Diego, CA, USA). Gametocyte activation, percentage of female/male gametocyte-forming gametes, exflagellation center formation, and ookinete conversion rate were compared by Student’s t-test. The prevalence of infection (proportion of infected mosquitoes) was analyzed by the Fisher’s exact test (with Bonferroni correction), while the oocyst density (oocyst number per midgut) was analyzed by the Mann–Whitney U-test. A *P*-value < 0.05 was considered to be statistically significant.

## Results

### Expression of recombinant proteins

**Fig. 1:**
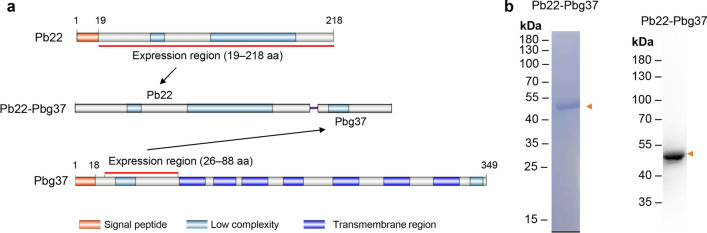
Expression and purification of rPbg37-Pb22 fusion protein and antibody response in mice immunized with rPbg37, rPb22, rPbg37-Pb22 and rPbg37+Pb22. **a** Expressed regions of the Pbg37 (26–88 aa), Pb22 (19–218 aa) and Pbg37-Pb22 (Pbg37^26–88 aa^-Pb22^19–218 aa^) fusion protein. The signal peptide (red box), low complexity (cyan box) and transmembrane region (blue box) are highlighted. The pink line denotes the linker. **b** Analysis of purified recombinant Pbg37-Pb22 highlighted by Coomassie blue staining (left panel) and on Western blot (anti-His mAb, right panel). Red arrowheads indicate the target rPbg37-Pb22 protein band. aa, Amino acid; mAb, monoclonal antibody; Pb22, *Plasmodium berghei* 22 antigen; Pbg37, *P. berghei* G37 antigen; rPbg37-Pb22, recombinant Pbg37-Pb22 fusion protein; rPbg37+Pb22, mixed recombinant Pbg37 and Pb22 protein

To explore the immunogenicity of a dual-antigen vaccine targeting Pb22 and Pbg37, we generated a chimeric construct containing the His_20_-Ser_218_ fragment of Pb22 fused to the Gln_27_-Asn_88_ fragment of Pbg37 using a flexible linker sequence (GGGGS)_3_ (Fig. 1a). Although the identity of the selected expression region of rPb22^20−218^ was only 34.7% among *P. berghei*, *P. falciparum* and *P. vivax*, the selected expression region of Pbg37^27−88^ is highly conserved (identity: 69.4%) among *Plasmodium* spp. (Additional file 2: Fig. S1a, b). The Pb22-Pbg37 fusion protein was induced in *E. coli* by 1 mM IPTG at 19 °C overnight and purified using Ni–NTA chromatography (Fig. 1a). SDS-PAGE analysis showed that the purified rPb22-Pbg37 construct was approximately 51 kDa (including the N-terminal Trx-His), consistent with its predicted molecular weight (Fig. 1b). The recombinant protein reacted with the anti-His mAb, confirming the specificity of the generated chimeric protein (Fig. 1b). In addition, rPb22 (His_20_-Ser_218_) and rPbg37 (Gln_27_-Asn_88_) were generated for parallel analysis. The purified rPb22 and rPbg37 had a molecular weight of approximately 43 and 26 kDa, respectively, which is in agreement with their predicted size (Additional file 3: Fig. S2a, b).

### The Pb22-Pbg37 fusion protein is immunogenic

**Fig. 2:**
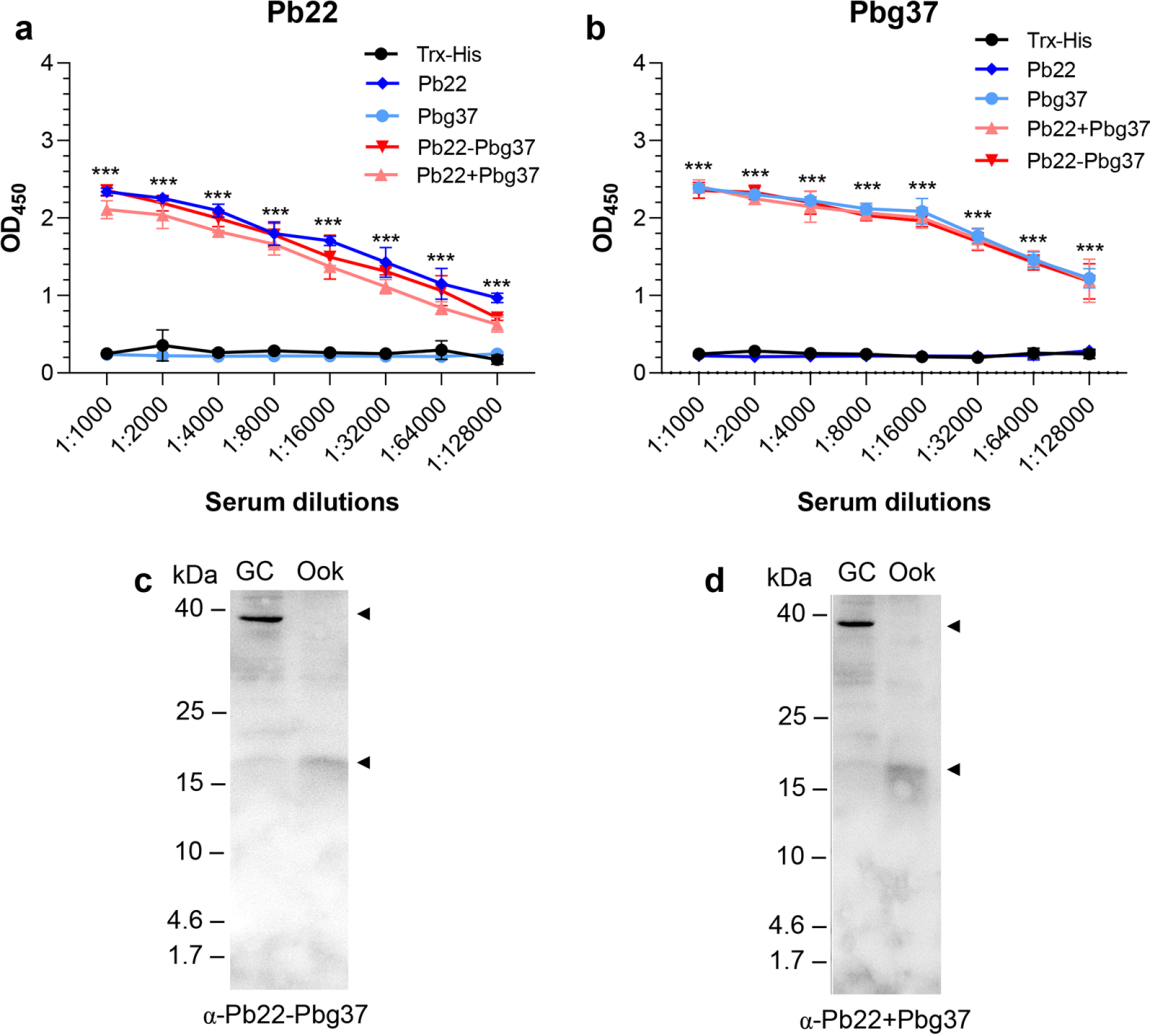
Immunogenicity of the Pb22-Pbg37 and Pb22+Pbg37 antigens. **a**, **b** Comparison of total IgG titers induced by the fused (Pb22-Pbg37) or mixed (Pb22+Pbg37) antigens and single antigens (Trx-His, Pb22 and Pbg37). The analyzed immunization sera of every mouse per group were pooled at day 10 after the final immunization for ELISA coated with the recombinant Pbg37 (**a**) and recombinant Pb22 (**b**) protein. Error bars indicate standard deviation. Asterisks indicate a significant difference between the immunization and control groups at ***P < 0.001 by Analysis of variance (Student’s t-test). **c**, **d** Western blot analysis under reducing conditions using the anti-Pb22-Pbg37 and anti-Pb22+Pbg37 sera on purified gametocyte and ookinete lysates of *Plasmodium berghei* ANKA parasites, respectively. Heat shock protein 70 (Hsp70) was used as loading control. GC, Non-activated gametocytes; Ook, ookinetes; Pb22, *Plasmodium berghei* 22 antigen; Pbg37, *P. berghei* G37 antigen; Pbg37-Pb22, Pbg37-Pb22 fusion protein; Pbg37+Pb22, mixed Pbg37 and Pb22 protein; Trx-His, negative control recombinant protein

To investigate the immunogenicity of the mixed (Pb22+Pbg37) and fused (Pb22-Pbg37) recombinant protein, we first determined the specific antibody responses induced by each of these antigens as compared with those induced by the corresponding single antigens (Trx-His Pb22 and Pbg37). Mice were immunized with a 2-week interval between the prime and each boost injection. As expected, immunization with the individual antigens yielded only antibodies specific to the respective antigen used for immunization. Although the antibody titers against Pb22 or Pbg37 induced by rPb22 or rPbg37 alone were higher than the antibody titer induced by Pb22 mixed/fused with Pbg37, the results were not significant, as determined in ELISA analysis using immune serum collected 2 weeks after the final immunization (Fig. 2a, b). In addition, the antibody titers of the Pb22+Pbg37 and Pb22-Pbg37 immunization groups were comparable, indicating that the mixed and fused antigens had a similar immunogenicity (Fig. 2a, b).

Although both Pb22 and Pbg37 are both pre- and post-fertilization-phase TBV candidates, Pb22 is dominantly expressed on the outer surface of ookinetes, whereas Pbg37 is expressed primarily in gametocytes and gametes and, to a lesser extent, in zygotes and ookinetes [17, 18]. To verify the reactivity of antisera raised against the mixed and fused recombinant proteins with the parasite native protein, we examined the cell lysates from gametocytes and ookinetes by western blot using antisera against Pb22-Pbg37 and Pb22+Pbg37, respectively. Both antisera against the mixed and fused antigen of Pb22 and Pbg37 identified a band of approximately 37 kDa and a faint band of approximately 25 kDa in gametocyte lysates, corresponding to the endogenous Pbg37 and Pb22 protein, respectively (Fig. 2c, d). In comparison, in the lysate of ookinetes, both anti-Pb22-Pbg37 and anti-Pb22+Pbg37 sera identified a band of approximately 25 kDa, corresponding to the Pb22 protein (Fig. 2c, d). Consistent with the previous report that the expression level of Pbg37 was reduced in the ookinete stage, we observed a band of approximately 37 kDa that corresponded to endogenous Pbg37 protein when the exposure time was extended to 3 min (data not shown).

### Reactivity of the antisera with the endogenous Pb22 and Pbg37 protein

**Fig. 3:**
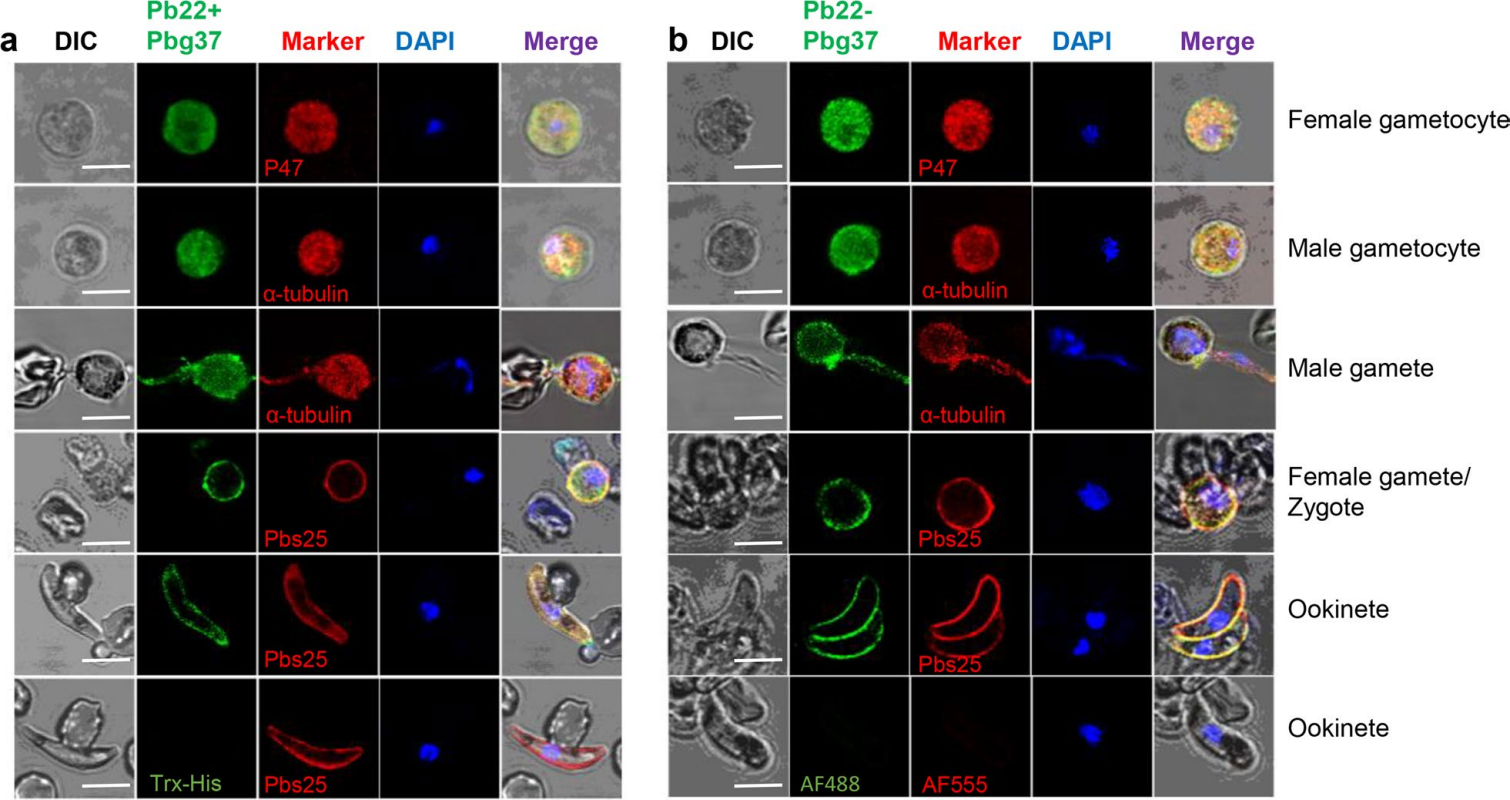
Immunofluorescence assays using the anti-Pb22-Pbg37 and anti-Pb22+Pbg37 sera. Parasites at different developmental stages were fixed and permeabilized with 0.1% Triton X-100, followed by staining with antisera against Pbg37+Pb22 (**a**) and Pbg37-Pb22 (**b**) (1:500) as the primary antibodies (green). The parasites were also co-labeled with antibodies against the markers specific for different developmental stages (red), including P47 for female gametocytes, α-tubulin for male gametocytes/gametes and Pbs25 for zygotes and ookinetes. Alexa Fluor 488-conjugated goat anti-mouse immunoglobulin G antibodies (IgG) and Alexa Fluor 555-conjugated goat anti-rabbit IgG antibodies were used as secondary antibodies. Ookinetes staining with anti-Trx-His sera or with the secondary antibodies only were used as negative controls. The nucleus was stained with DAPI (blue). Scale bar: 5 μm. DIC, Differential interference contrast microscopy; Pb22, *Plasmodium berghei* 22 antigen; Pbg37, *P. berghei* G37 antigen; Pbg37-Pb22, Pbg37-Pb22 fusion protein; Pbg37+Pb22, mixed Pbg37 and Pb22 protein; Trx-His, negative control recombinant protein

To further characterize the specificity of the antibodies, an IFA of the parasite at different sexual stages was performed. Both antisera (anti-Pb22-Pbg37 and anti-Pb22+Pbg37 sera) against the mixture (Pb22+Pbg37) and fused antigen (Pb22-Pbg37) reacted with the gametocytes, gametes, zygotes and ookinetes (Fig. 3a, b). Comparable to the location pattern of Pbg37 and Pb22 observed in previously reported studies [17, 18], we found that the fluorescence was mostly associated with the residual body and flagella in the exflagellating male gametes. When detected by anti-Pb22-Pbg37 and anti-Pb22+Pbg37 sera, we observed strong fluorescence signals surrounding the plasma membranes of female gametes/zygotes and ookinetes, indicating an abundant surface localization pattern of Pb22 and Pbg37 (Fig. 3a, b). In contrast, no fluorescence signals were observed in negative controls of ookinetes using anti-Trx-His sera or using the secondary antibodies alone (Fig. 3a, b).

### Antisera against fusion and mixed dual antigens improve TRA

**Fig. 4:**
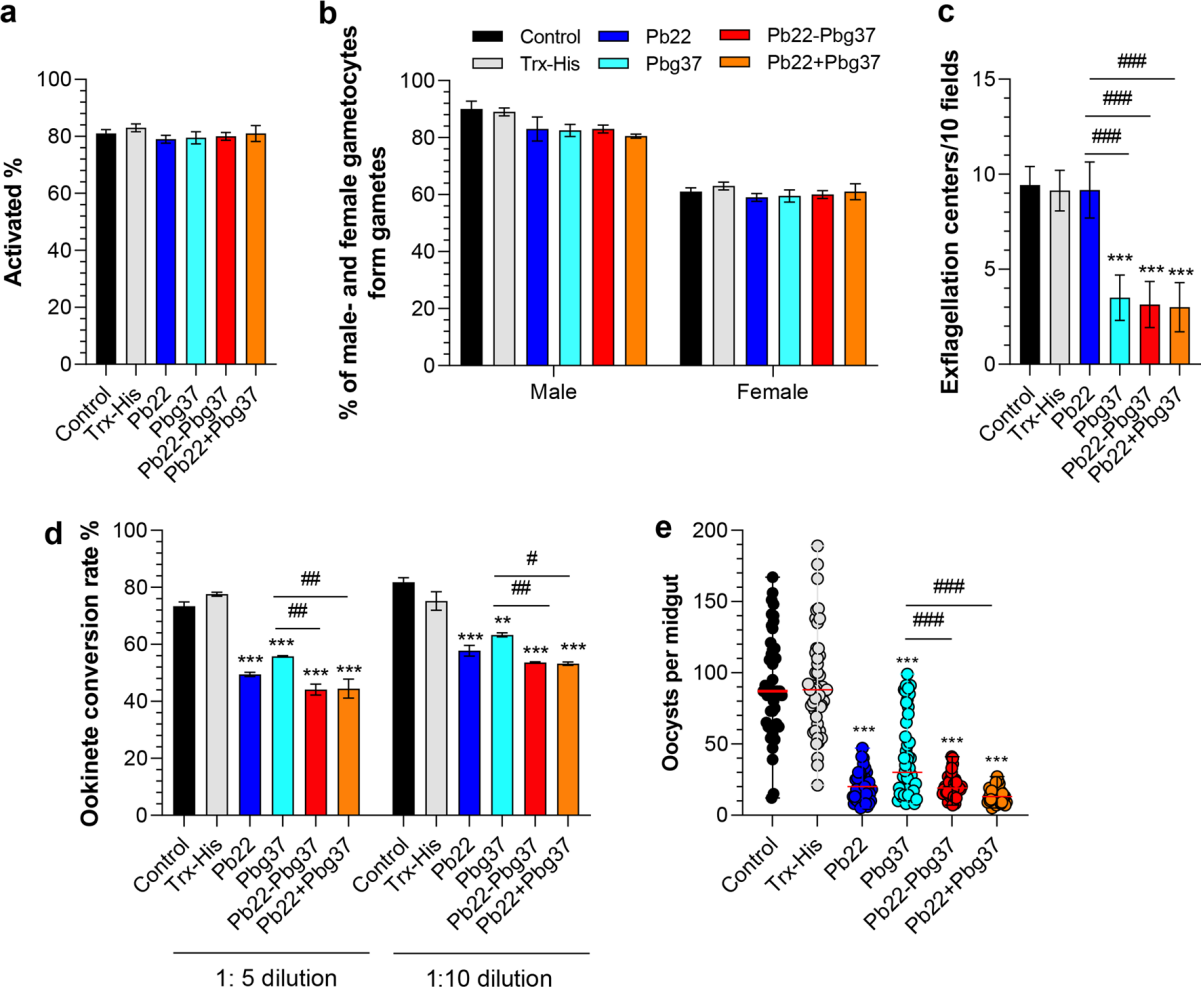
*Plasmodium berghei* transmission blocking with anti-Pb22-Pbg37 and anti-Pb22+Pbg37 sera. *Plasmodium berghei*-infected blood collected at 3 days post-infection was incubated with the respective control (pre-immune sera [control] and anti-Trx-His sera) and immune sera (anti-Pbg37, anti-Pb22, anti-Pbg37-Pb22 and anti-Pbg37+Pb22) at dilutions of 1:5, and the inhibition of gametocyte activation (**a**), male-/female gametocyte-forming gametes (**b**) and exflagellation (**c**) was determined after 10 min (gametocyte activation and gamete formation) and 15 min (exflagellation center formation), respectively. **d** Inhibition of ookinete conversion. The ookinete conversion rate at 24 h post-in vitro culture determined by staining with Pbs21 monoclonal antibody and counted. Data for gametocyte activation, gamete formation exflagellation center formation, and ookinete conversion rate are representative of three independent experiments. **e** Transmission-blocking activity of anti-Pb22-Pbg37 and anti-Pb22+Pbg37 sera on *P. berghei* oocyst numbers in mosquito midgut. Error bars indicate mean ± standard deviation. Mann–Whitney *U* test was used for statistical analysis of the oocyst density. The gametocyte activation, male-/female gametocyte-forming gametes, exflagellation centers and ookinete conversion were analysed by analysis of variance (ANOVA). Asterisks indicate a significant difference at ** *P* < 0.01 and ***P < 0.001 between the respective immunization group and the Trx-His control group; hashtags indicate a significant difference at # *P* < 0.05, ## *P* < 0.01 and ###*P* < 0.001 between two immunization groups (as indicated). Pb22, *Plasmodium berghei* 22 antigen; Pbg37, *P. berghei* G37 antigen; Pbg37-Pb22, Pbg37-Pb22 fusion protein; Pbg37+Pb22, mixed Pbg37 and Pb22 protein; Trx-His, negative control recombinant protein

To determine whether antisera against mixed (Pb22+Pbg37) or fused (Pb22-Pbg37) dual antigens could improve TRA, these antisera were first assessed using in vitro gametocyte activation and male-/female-gamete formation assays. The results showed that immunization with these antisera were comparable to that of single antigen immunization, as well as to the Trx-His immunization and the non-immunized control (Fig. 4a, b). However, when *P. berghei* gametocytes were incubated with the immune sera, exflagellation of male gametocytes was inhibited by the antisera against Pbg37, Pb22-Pbg37 and Pb22+Pbg37 by 60.9%, 65.6% and 67.2%, respectively, at 1:5 dilution, as compared to the Trx-His immunization group (Fig. 4d). In contrast, antisera against Pb22 antigen alone showed no significant impacts on the formation of exflagellation centers (Fig. 4c).

The ookinete conversion rates of the antisera against Pb22+Pbg37 and Pb22-Pbg37 at 1:5 dilution were 44.2 and 44.5, corresponding to a reduction of ookinete formation of 43.1% and 42.7%, respectively, compared to the Trx-His group. In comparison, antisera against single antigens Pbg37 and Pb22 reduced ookinete conversion rates by 28.1% and 36.3%, respectively. A similar trend of reduction was observed in vitro ookinete conversion rates when these antisera were used at 1:10 dilution. The ookinete conversion rates with the antisera against Pb22-Pbg37 (53.7) and Pb22+Pbg37 (53.2) were significantly larger than the rates at 1:5 dilutions (Fig. 4d). This result showed that the sera against mixed and fused antigens produced stronger inhibition effects on ookinete formation than the antisera against individual antigens and that the effects of the immune sera against the mixed and fused antigens on ookinete conversion rates were in an antisera dose-dependent manner.

We then investigated whether the fused and mixed antisera could inhibit oocyst development in the mosquito midgut. We passively transferred the antisera against fused or mixed antigens to mice infected with *P. berghei* before direct mosquito feeding and analyzed oocyst development in the mosquito midgut at 10 dpi. A statistically significant reduction in oocyst numbers (56.2–85.3% reduction) present in the mosquito midgut at 10 days post-direct feeding was observed for all immunization groups tested (Pb22, Pbg37, Pb22-Pbg37 and Pb22+Pbg37), compared to the Trx-His immunization control (*P* < 0.001, Mann–Whitney U-test; Fig. 4e; Table1). Furthermore, there were significant reductions in oocyst density in the mixed-antigen (85.3%) and fused-antigen (78.4%) immunization groups compared with the Pbg37 (56.2%) immunization groups (*P* < 0.001, Mann–Whitney U-test; Fig. 4e; Table1). In comparison, the Pb22 alone immunization group exhibited comparable reduction in oocyst density (77.3%) compared to the mixed antigen and fused antigen groups (Fig. 4e; Table 1). Meanwhile, the mosquito infection prevalence in all immunization groups was also reduced by 6–24%, compared to the Trx-His immunization control (Table1). Collectively, the mosquito feeding assay demonstrated that the Pb22 is a promising TBV target for blocking mosquito stage and that mixed or fused Pb22 and Pbg37 produced significantly higher TRA than did Pbg37 used alone.

**Table 1:** Transmission-blocking activity of mixed or fused Pb22 and Pbg37 antigen-immunized groups

Immunization groups	Oocyst density mean (range) (*n* = 50)	Transmission-reducing activity^a^ (%)	Prevalence of infection, mean (*n* = 50)	Transmission-blocking activity (%)
Control	90.8 (12–167)		94% (47/50)	
Trx-His	91.8 (21–189)		98% (49/50)	
Pb22	20.2 (5–47)	77.3%	82% (41/50)	16%
Pbg37	40.2 (8–99)	56.2%	92% (46/50)	6%
Pb22-Pbg37	20.5 (7–41)	78.4%	74% (37/50)	24%
Pb22+Pbg37	13.5 (5–27)	85.3%	76% (38/50)	22%

*Pb22* *Plasmodium berghei* 22 antigen,* Pbg37* *P. berghei* G37 antigen,* Pbg37-Pb22* Pbg37-Pb22 fusion protein,* Pbg37+Pb22* mixed Pbg37 and Pb22 protein,* Trx-His* negative control recombinant protein

^a^Transmission-reducing activity was calculated as (mean oocyst density_Trx-His_ − mean oocyst density_Pb22/Pgb37/Pb22-Pgb37/Pb22+Pbg37_)/(mean oocyst density_Trx-His_) × 100%

^b^Transmission-blocking activity was calculated as % prevalence_Trx-His_ − % prevalence_Pb22/Pgb37/Pb22-Pgb37/Pb22+Pbg37_

## Discussion

In a previous study we identified Pbg37 as a conserved antigen in *Plasmodium* spp. and essential for the male gamete [18]. Although Pbg37 is a candidate TBV antigen expressed in both pre- and post-fertilization stages, its expression level was observed to gradually decrease during the transition from zygote to ookinete [18]. Consistent with this observation, we also found that the anti-Pbg37 antisera generated against the N-terminal extracellular region of Pbg37 could decrease the formation of exflagellation centers by 70% at 1:5 dilution, but only slightly affected the ookinete conversion rate [18]. These results indicate that Pbg37 is a more efficient TBV antigen for the pre-fertilization stages (gametes) than post-fertilization stages (ookinetes). In contrast, knockout of the candidate TBV antigen Pb22 also expressed in the pre- and post-fertilization phase caused a severe male-specific deficiency (reduced formation of exflagellation centers by approx. 89%), reduced ookinete formation by approximately 97% and completely blocked oocyst formation in Δpb22 parasites [17]. Meanwhile, in that same study, the anti-Pb22 sera showed a concentration-dependent TBA, with a reduction in exflagellation center formation by 56.3%, a 83.3%-93.3% reduction in ookinete number and up to a 93.3% and 99.6% reduction in the prevalence of infected mosquitoes and oocyst density [17]. This excellent TBA of Pb22 during specific stages of mosquito development suggests that Pb22 might be a more promising target for selection as a post-fertilization TBV antigen. Because natural antibodies against pre-fertilization antigens can be detected in mosquito populations found in malaria endemic areas, immunization with these antigens could offer the advantage of boosting, while the expression of post-fertilization antigen could extend the time of TBA. Therefore, antigens such as Pb22 and Pbg37, which are expressed in both pre- and post-fertilization stages, may induce stronger TBAs.

A single vaccine expressing two antigens could potentially increase both the size and breadth of the antigen-specific response while halving vaccine production costs. Therefore, we sought to increase TBA by combining the two TBV candidates Pb22 and Pbg37. A previous in silico analysis showed that the (GGGGS)_3_ linker confers a better structure and stability for the fusion protein [35]; consequently, we selected this linker in our current study for evaluating the efficiency of our fusion protein Pb22-Pbg37. Despite theoretical concerns that the administration of more than one antigen would result in antigenic competition [36, 37], no detrimental effect was observed when any of Pb22 and Pbg37 antigens were co-administered. The administration of Pb22 fused or mixed with Pbg37 showed a significantly higher antibody titer compared to the Trx-His control group. Meanwhile, the antibody titers against Pb22-Pbg37 and Pb22+Pbg37 were similar to each other, suggesting that either mixing or fusion of these two antigens is comparable in terms of immunogenicity. Furthermore, the anti-sera raised against both the mixed and fusion candidate TBV antigens were able to recognize the respective endogenous proteins expressed from gametocyte to ookinete stages during sexual-stage development. Together, these results suggest that combining the Pb22 and Pbg37 antigens of the malaria parasite did not result in substantial immune interference.

Although the polyclonal antibody titer was found to be higher in the groups immunized with Pb22 and Pbg37 alone compared with the groups immunized with the fusion (Pb22-Pbg37) or mixed (Pb22+Pbg37) antigen, it did not result in a higher protection. Vaccination with a combination of Pb22 and Pbg37, either fused or mixed, was shown to improve protective efficacy compared to vaccination with each single antigen alone. The efficacy of vaccination with fusion-antigen (Pb22-Pbg37) was equivalent to that of vaccination with a mixture of the single antigens (Pb22+Pbg37). Similar phenomena have also been reported in pre-erythrocyte-stage fusion antigen studies, such as combining immunization with circumsporozoite protein (CSP) with either of the non-CSP pre-erythrocyte-stage antigens [38]. For pre-erythrocyte antigen immunization, the lack of correlation between antibody titer and protective immunity may be caused by the CD8^+^ T cell-mediated cellular immune response [39, 40]. In our study, it is possible that the functional antibodies within the antisera were a mixture against both on-target specific epitopes and off-target cross-reactivity; such mixtures have also been detected in some preclinical studies of RTS,S vaccine [41–44]. In addition, the Pb22 and Pbg37 dual-antigen-immunized mice may provide a better TBA than anti-immune sera passively transferred to mice, which could be ruled out in our future studies. Therefore, a more extensive study is required to investigate whether the specific protective epitopes would be valuable. It is also important to characterize the antibody responses that are directly associated with protection, such as avidity, antibody maturation and IgG subclass.

## Conclusions

Anti-malarial TBV that target the surface of the gamete and ookinete stages of *Plasmodium* inhibit further development of the parasite within the mosquito host and, therefore, have a significant efficiency for reducing malaria transmission in endemic areas. Our data confirm the previous finding that both Pb22 and Pbg37 are promising TBV candidates. The data described here add to previously presented data showing the significant potential of developing antisera (or antibodies) against dual antigens (Pb22 and Pbg37) as malaria vaccine candidates, thereby addressing the urgent need for effective vaccines capable of intervening in malaria parasite transmission.

### Supplementary Information

**Additional file 1: Table S1.** Primers used in this study.

**Additional file 2: Figure S1.** Sequence alignment of Pb22 and Pbg37 in *Plasmodium* spp. Pb, *Plasmodium berghei*; Pf, *Plasmodium falciparum*; Pv, *Plasmodium vivax*. Green boxes indicate the regions used for generating the Pb22 and Pbg37 recombinant proteins.

**Additional file 3: Figure S2.** Recombinant protein expression of Pb22 and Pbg37 using the *Escherichia coli *system. Purified recombinant Pb22 (**a**) and Pbg37 (**b**) were separated on a 10% SDS-PAGE gel and stained with Coomassie blue (left) and probed with anti-His tag antibody for immunoblot assays (right), respectively. Arrows indicate the expressed recombinant Pb22 and Pbg37 protein, respectively.

## Data Availability

The data supporting the conclusions of this article are included within the article.

## Abbreviations

BSA: Bovine serum albumin
DFA: Direct mosquito feeding assay
dpi: Days post-infection
ELISA: Enzyme-linked immunosorbent assay
IFA: Immunofluorescence assay
mAb: Monoclonal antibody
PHZ: Phenylhydrazine
PVDF: Polyvinylidene fluoride
SDS-PAGE: Sodium dodecyl sulfate–polyacrylamide gel electrophoresis
TBA: Transmission-blocking activity
TBV: Transmission-blocking vaccine
TMB: Tetramethylbenzidine
TRA: Transmission-reducing activity

## References

[1] WHO. World malaria report 2021. 2022. https://www.who.int/teams/global-malaria-programme/reports/world-malaria-report-2021. Accessed 2023.

[2] Dondorp AM, Nosten F, Yi P, Das D, Phyo AP, Tarning J (2009). Artemisinin resistance in *Plasmodium falciparum* malaria. N Engl J Med.

[3] Laurens MB (2020). RTS,S/AS01 vaccine (Mosquirix): an overview. Hum Vaccin Immunother.

[4] White MT, Verity R, Churcher TS, Ghani AC (2015). Vaccine approaches to malaria control and elimination: insights from mathematical models. Vaccine.

[5] Alonso PL, Brown G, Arevalo-Herrera M, Binka F, Chitnis C, Collins F (2011). A research agenda to underpin malaria eradication. PLoS Med.

[6] malERA Consultative Group on Drugs (2011). A research agenda for malaria eradication: drugs. PLoS Med.

[7] Carter R, Chen DH (1976). Malaria transmission blocked by immunisation with gametes of the malaria parasite. Nature.

[8] Gwadz RW (1976). Successful immunization against the sexual stages of *Plasmodium gallinaceum*. Science.

[9] Stokes BH, Ward KE, Fidock DA (2022). Evidence of artemisinin-resistant malaria in Africa. N Engl J Med.

[10] Takashima E, Tachibana M, Morita M, Nagaoka H, Kanoi BN, Tsuboi T (2021). Identification of novel malaria transmission-blocking vaccine candidates. Front Cell Infect Microbiol.

[11] Marin-Mogollon C, van de Vegte-Bolmer M, van Gemert GJ, van Pul FJA, Ramesar J, Othman AS (2018). The *Plasmodium falciparum* male gametocyte protein P230p, a paralog of P230, is vital for ookinete formation and mosquito transmission. Sci Rep.

[12] MacDonald NJ, Nguyen V, Shimp R, Reiter K, Herrera R, Burkhardt M (2016). Structural and immunological characterization of recombinant 6-cysteine domains of the *Plasmodium falciparum* sexual stage protein Pfs230. J Biol Chem.

[13] Theisen M, Jore MM, Sauerwein R (2017). Towards clinical development of a Pfs48/45-based transmission blocking malaria vaccine. Expert Rev Vaccines.

[14] Blagborough AM, Sinden RE (2009). *Plasmodium berghei* HAP2 induces strong malaria transmission-blocking immunity in vivo and in vitro. Vaccine.

[15] Qiu Y, Zhao Y, Liu F, Ye B, Zhao Z, Thongpoon S (2020). Evaluation of *Plasmodium vivax* HAP2 as a transmission-blocking vaccine candidate. Vaccine.

[16] Saxena AK, Wu Y, Garboczi DN (2007). *Plasmodium* p25 and p28 surface proteins: potential transmission-blocking vaccines. Eukaryot Cell.

[17] Liu F, Yang F, Wang Y, Hong M, Zheng W, Min H (2021). A conserved malaria parasite antigen Pb22 plays a critical role in male gametogenesis in *Plasmodium berghei*. Cell Microbiol.

[18] Liu F, Li L, Zheng W, He Y, Wang Y, Zhu X, et al. Characterization of* Plasmodium berghei *Pbg37 as both a pre- and postfertilization antigen with transmission-blocking potential. Infect Immun. 2018;86:e00785-17. 10.1128/IAI.00785-17. 10.1128/IAI.00785-17 PMC6056874 29866905

[19] Kou X, Zheng W, Du F, Liu F, Wang M, Fan Q (2016). Characterization of a *Plasmodium berghei* sexual stage antigen PbPH as a new candidate for malaria transmission-blocking vaccine. Parasit Vectors.

[20] Wang PP, Jiang X, Bai J, Yang F, Yu X, Wu Y (2022). Characterization of PSOP26 as an ookinete surface antigen with improved transmission-blocking activity when fused with PSOP25. Parasit Vectors.

[21] Wang PP, Jiang X, Zhu L, Zhou D, Hong M, He L (2022). A G-protein-coupled receptor modulates gametogenesis via PKG-mediated signaling cascade in *Plasmodium berghei*. Microbiol Spectr..

[22] Wang J, Zheng W, Liu F, Wang Y, He Y, Zheng L (2017). Characterization of Pb51 in *Plasmodium berghei* as a malaria vaccine candidate targeting both asexual erythrocytic proliferation and transmission. Malar J.

[23] Zheng W, Liu F, Du F, Yang F, Kou X, He Y (2020). Characterization of a sulfhydryl oxidase from *Plasmodium berghei* as a target for blocking parasite transmission. Front Cell Infect Microbiol.

[24] Bai J, Liu F, Yang F, Zhao Y, Jia X, Thongpoon S (2023). Evaluation of transmission-blocking potential of Pv22 using clinical *Plasmodium vivax* infections and transgenic *Plasmodium berghei*. Vaccine.

[25] Baptista BO, de Souza ABL, Riccio EKP, Bianco-Junior C, Totino PRR, Martins da Silva JH (2022). Naturally acquired antibody response to a *Plasmodium falciparum* chimeric vaccine candidate GMZ26c and its components (MSP-3, GLURP, and Pfs48/45) in individuals living in Brazilian malaria-endemic areas. Malar J.

[26] Mistarz UH, Singh SK, Nguyen T, Roeffen W, Yang F, Lissau C (2017). Expression, purification and characterization of GMZ2'.10C, a complex disulphide-bonded fusion protein vaccine candidate against the asexual and sexual life-stages of the malaria-causing plasmodium falciparum parasite. Pharm Res.

[27] Collins KA, Snaith R, Cottingham MG, Gilbert SC, Hill AVS (2017). Enhancing protective immunity to malaria with a highly immunogenic virus-like particle vaccine. Sci Rep.

[28] Yusuf Y, Yoshii T, Iyori M, Mizukami H, Fukumoto S, Yamamoto DS (2019). A viral-vectored multi-stage malaria vaccine regimen with protective and transmission-blocking efficacies. Front Immunol.

[29] Mizutani M, Iyori M, Blagborough AM, Fukumoto S, Funatsu T, Sinden RE (2014). Baculovirus-vectored multistage *Plasmodium vivax* vaccine induces both protective and transmission-blocking immunities against transgenic rodent malaria parasites. Infect Immun.

[30] Yang F, Liu F, Yu X, Zheng W, Wu Y, Qiu Y (2021). Evaluation of two sexual-stage antigens as bivalent transmission-blocking vaccines in rodent malaria. Parasit Vectors.

[31] Miura K, Orcutt AC, Muratova OV, Miller LH, Saul A, Long CA (2008). Development and characterization of a standardized ELISA including a reference serum on each plate to detect antibodies induced by experimental malaria vaccines. Vaccine.

[32] Tonkin CJ, van Dooren GG, Spurck TP, Struck NS, Good RT, Handman E (2004). Localization of organellar proteins in *Plasmodium falciparum* using a novel set of transfection vectors and a new immunofluorescence fixation method. Mol Biochem Parasitol.

[33] Tewari R, Straschil U, Bateman A, Bohme U, Cherevach I, Gong P (2010). The systematic functional analysis of *Plasmodium* protein kinases identifies essential regulators of mosquito transmission. Cell Host Microbe.

[34] Yoshida S, Matsuoka H, Luo E, Iwai K, Arai M, Sinden RE (1999). A single-chain antibody fragment specific for the *Plasmodium berghei* ookinete protein Pbs21 confers transmission blockade in the mosquito midgut. Mol Biochem Parasitol.

[35] Shamriz S, Ofoghi H, Moazami N (2016). Effect of linker length and residues on the structure and stability of a fusion protein with malaria vaccine application. Comput Biol Med.

[36] Sheehy SH, Duncan CJ, Elias SC, Choudhary P, Biswas S, Halstead FD (2012). ChAd63-MVA-vectored blood-stage malaria vaccines targeting MSP1 and AMA1: assessment of efficacy against mosquito bite challenge in humans. Mol Ther.

[37] Elias SC, Collins KA, Halstead FD, Choudhary P, Bliss CM, Ewer KJ (2013). Assessment of immune interference, antagonism, and diversion following human immunization with biallelic blood-stage malaria viral-vectored vaccines and controlled malaria infection. J Immunol.

[38] Vigdorovich V, Patel H, Watson A, Raappana A, Reynolds L, Selman W (2023). Coimmunization with preerythrocytic antigens alongside circumsporozoite protein can enhance sterile protection against *Plasmodium* Sporozoite infection. Microbiol Spectr.

[39] Meraldi V, Romero JF, Kensil C, Corradin G (2005). A strong CD8+ T cell response is elicited using the synthetic polypeptide from the C-terminus of the circumsporozoite protein of *Plasmodium berghei* together with the adjuvant QS-21: quantitative and phenotypic comparison with the vaccine model of irradiated sporozoites. Vaccine.

[40] Moris P, Jongert E, van der Most RG (2018). Characterization of T-cell immune responses in clinical trials of the candidate RTS S malaria vaccine. Hum Vaccin Immunother.

[41] Kurtovic L, Atre T, Feng G, Wines BD, Chan JA, Boyle MJ (2021). Multifunctional antibodies are induced by the RTS, S malaria vaccine and associated with protection in a phase 1/2a trial. J Infect Dis.

[42] Mugo RM, Mwai K, Mwacharo J, Shee FM, Musyoki JN, Wambua J (2021). Seven-year kinetics of RTS,S/AS01-induced anti-CSP antibodies in young Kenyan children. Malar J.

[43] Dobano C, Sanz H, Sorgho H, Dosoo D, Mpina M, Ubillos I (2019). Concentration and avidity of antibodies to different circumsporozoite epitopes correlate with RTS,S/AS01E malaria vaccine efficacy. Nat Commun.

[44] Macia D, Campo JJ, Moncunill G, Jairoce C, Nhabomba AJ, Mpina M (2022). Strong off-target antibody reactivity to malarial antigens induced by RTS,S/AS01E vaccination is associated with protection. JCI Insight.

