# Supplementary figures and images for: A dual-antigen malaria vaccine targeting Pb22 and Pbg37 was able to induce robust transmission-blocking activity
Source: Parasit Vectors. 2023 Dec 14;16:455. doi: 10.1186/s13071-023-06071-x (PMC10720250; doi:10.1186/s13071-023-06071-x)

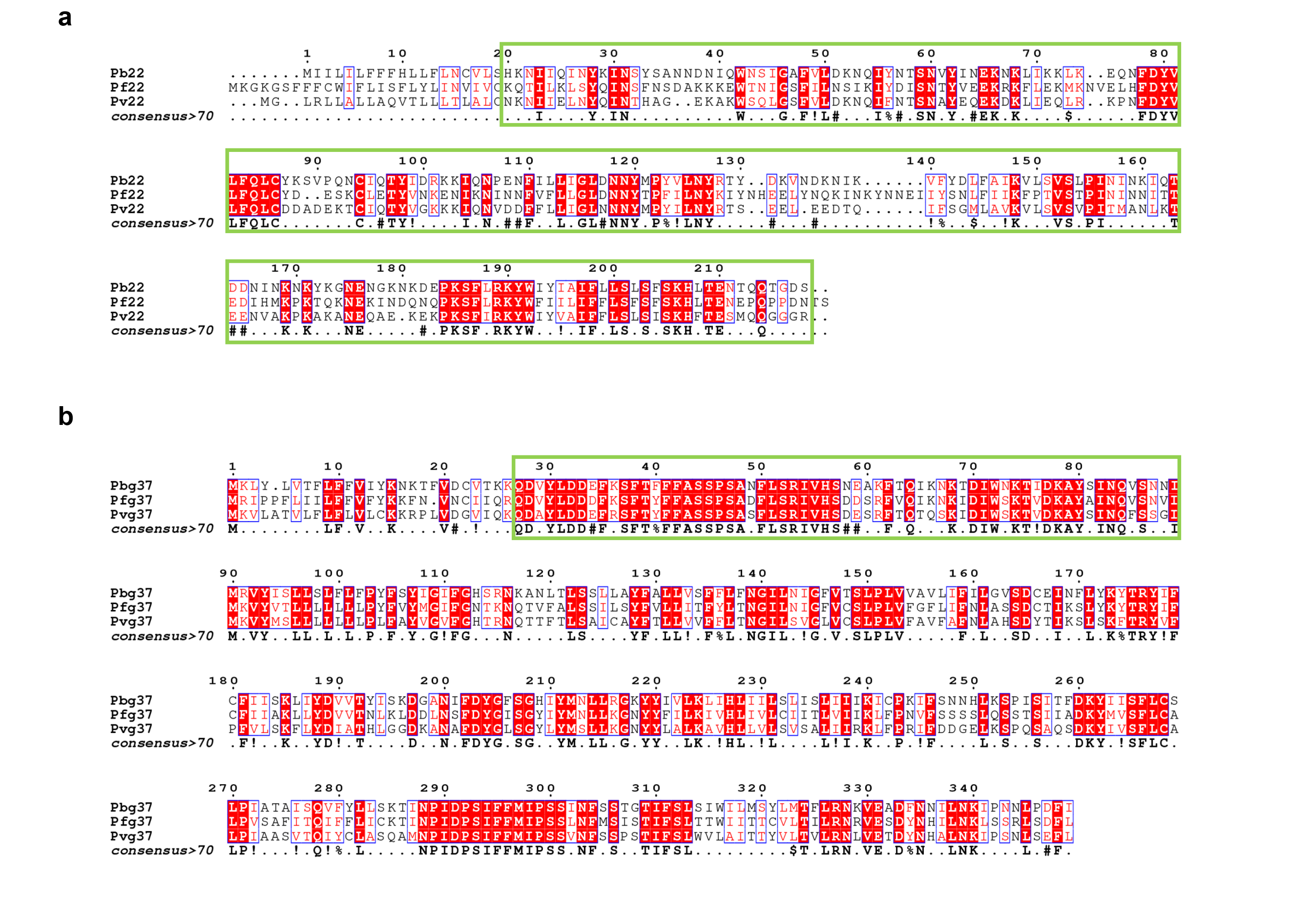

Supplement: Supplementary file 1 — Additional file 1: Table S1. Primers used in this study. [file 13071_2023_6071_MOESM1_ESM.tif]

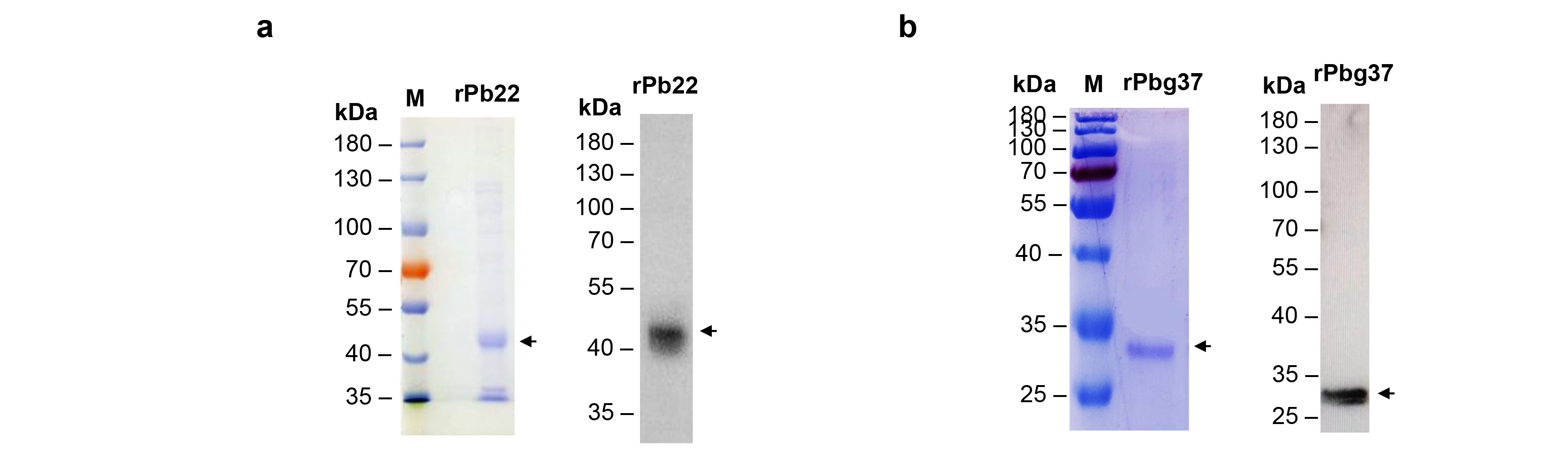

Supplement: Supplementary file 2 — Additional file 2: Figure S1. Sequence alignment of Pb22 and Pbg37 in Plasmodium spp. Pb, Plasmodium berghei; Pf, Plasmodium falciparum; Pv, Plasmodium vivax. Green boxes indicate the regions used for generating the Pb22 and Pbg37 recombinant proteins. [file 13071_2023_6071_MOESM2_ESM.tif]
