# Supplementary material for: A dual-antigen malaria vaccine targeting Pb22 and Pbg37 was able to induce robust transmission-blocking activity
Source: Parasit Vectors. 2023 Dec 14;16:455. doi: 10.1186/s13071-023-06071-x (PMC10720250; doi:10.1186/s13071-023-06071-x)
Supplement: Supplementary file 3 — Additional file 3: Figure S2. Recombinant protein expression of Pb22 and Pbg37 using the Escherichia coli system. Purified recombinant Pb22 (a) and Pbg37 (b) were separated on a 10% SDS-PAGE gel and stained with Coomassie blue (left) and probed with anti-His tag antibody for immunoblot assays (right), respectively. Arrows indicate the expressed recombinant Pb22 and Pbg37 protein, respectively. [file 13071_2023_6071_MOESM3_ESM.docx]

**Table S1. Primers information and sequence**

| Primer | Sequence |
| --- | --- |
| Pb22-F | CTGGATCCTCACATAAAAATATAATTCAAATAAATTAT |
| Pb22-R-Linker | ACCGCCGCTTCCTCCGCCTCCGCTTCCGCCTCCGCCTTATGAATCACCTGTCTGTTGTGTATTTTCA |
| Linker-pbg37-F | GGAGGCGGAGGAAGCGGCGGTGGCGGCAGC AAACAGGATGTTTATTTGGATGAT |
| Pbg37-R | CAGCGGCCGCTTAATTATTTGAAACCTGATTAATTGAGT |
